# Polyunsaturated fatty acids stimulate immunity and eicosanoid production in *Drosophila melanogaster*

**DOI:** 10.1016/j.jlr.2024.100608

**Published:** 2024-07-26

**Authors:** Pakeeza Azizpor, Ogadinma K. Okakpu, Sophia C. Parks, Diego Chavez, Fayez Eyabi, Stephanie Martinez-Beltran, Susan Nguyen, Adler R. Dillman

**Affiliations:** Department of Nematology, University of California, Riverside, CA, USA

**Keywords:** Drosophila, arachidonic acid, linoleic acid, hemolymph, lipidomics

## Abstract

Eicosanoids are a class of molecules derived from C20 polyunsaturated fatty acids (PUFAs) that play a vital role in mammalian and insect biological systems, including development, reproduction, and immunity. Recent research has shown that insects have significant but lower levels of C20 PUFAs in circulation in comparison to C18 PUFAs. It has been previously hypothesized in insects that eicosanoids are synthesized from C18 precursors, such as linoleic acid (LA), to produce downstream eicosanoids. In this study, we show that introduction of arachidonic acid (AA) stimulates production of cyclooxygenase, lipoxygenase, and cytochrome P450–derived eicosanoids. Downstream immune readouts showed that LA stimulates phagocytosis by hemocytes, while both LA and AA stimulate increased antimicrobial peptide production when *D. melanogaster* is exposed to a heat-killed bacterial pathogen. In totality, this work identifies PUFAs that are involved in insect immunity and adds evidence to the notion that *Drosophila* utilizes immunostimulatory lipid signaling to mitigate bacterial infections. Our understanding of immune signaling in the fly and its analogies to mammalian systems will increase the power and value of *Drosophila* as a model organism in immune studies.

Polyunsaturated fatty acids (PUFAs) play crucial roles in the development, regulation of bodily functions, and immunity across different organisms. PUFAs are broadly divided into two types: omega-3 (n*-3*) fatty-acids, which originate from α-linolenic acid (ALA), and omega-6 (n-6) fatty-acids which are obtained from linoleic acid (LA) (1). In mammals, PUFAs serve as substrates for three major lipid biosynthesis pathways, leading to the formation of eicosanoids. Upon hydrolysis from the membrane by phospholipase A_2_ (PLA_2_), eicosanoid precursors such as arachidonic acid (AA), eicosapentaenoic acid, and docosahexaenoic acid can be converted into prostaglandins (PGs) by cyclooxygenases (COXs), leukotrienes and hydroxyeicosatetraenoic acids (HETEs) by lipoxygenases (LOXs), and epoxyeicosatrienoic acid (EET), dihydroxyeicosatrienoic acids (diHETrE) and HETEs by cytochrome P450 (CYP450) (2, 3, 4, 5, 6, 7). In humans and animals, dietary imbalance in the ratios of n-6/n-3 PUFAs could adversely influence health by promoting the pathogenesis of many diseases, including cardiovascular disease, cancer, and inflammatory and autoimmune diseases (8). PUFAs play an important role in the composition of all cell membranes where they maintain homeostasis for correct membrane protein function and influence membrane fluidity, thus regulating cell signaling processes, cellular functions, and gene expression (9). PUFAs along with maintaining cell structure and function also enhance immune functions, promote growth and maturity, and regulate lipid metabolism and related gene expression.

In insects, it has been suggested that endogenous PLA_2_s cleave LA which can be converted into AA by desaturases and long-chain fatty acid elongase (10, 11). While previous studies suggested that insects lacked C20 PUFAs, new research showed that C20 PUFAs were able to be detected in significant concentrations in *Drosophila melanogaster* (12, 13). Although the overall research on eicosanoids in insects is relatively limited, it has been demonstrated that they play significant biological roles. For instance, PGs have been found to affect the egg-laying behavior of crickets, such as *Teleogryllus commodus*, influencing their reproduction (14, 15). In silk moths, inhibition of PG synthesis interferes with follicle development, and products of LOX and COX in *Rhodnius plexus*' ovaries regulate the uptake of *Rhodnius* heme-binding protein (16, 17, 18). Similarly, in ticks, PGE_2_ influences fluid secretion rates and composition, with inhibition of PLA_2_ and COX resulting in decreased fluid secretion and stimulation of salivary glands with PGE_2_ leading to various physiological responses (15, 16).

In humans, eicosanoids are well-known as lipid mediators involved in immune responses. Interestingly, research suggests that eicosanoids also participate in cellular and humoral immune responses in insects. The role of eicosanoids in insect immunity was first discovered in *Manduca sexta*, where inhibition of PLA_2_ decreased the organism's ability to clear bacteria from the hemolymph, a phenotype rescued by the addition of AA (17). Further investigations into insect immune responses revealed that cellular immune markers, such as nodulation and melanotic encapsulation, were influenced by eicosanoids (18, 19, 20). In addition, PUFAs also demonstrate bactericidal effects directly on gram-positive bacteria (21, 22).

*Drosophila melanogaster* fruit flies serve as a valuable model organism for studying disease. Fruit flies possess physical barriers and innate immunity, including cellular and humoral components. Cellular immunity involves melanization, phagocytosis, and encapsulation, while humoral immunity leads to the production of antimicrobial peptides (AMPs) (23, 24). Melanization occurs after production of phenoloxidase (PO) from upregulation of prophenoloxidase (25, 26). PO acts as a catalyst for melanization via mediation of oxidation of monophenols and diphenols to quinones, which after polymerization, forms antimicrobial melanin (27, 28). AMP production is regulated by two signaling pathways: The Toll pathway and the immune deficiency (Imd) pathway, which bear similarities to mammalian Toll-like receptor/interleukin 1 receptor signaling cascade and TNF-R pathway, respectively (23). The connection between PLA_2_-generated fatty acids and the lipopolysaccharide-activated Imd pathway has been established, with suppression of the Imd pathway observed when PLA_2_ inhibitors are used and rescued by the addition of AA (29). Initially, the absence of COX gene homologs within the genome of *D. melanogaster* raised intriguing questions about the synthesis of eicosanoids. However, a unique COX-like peroxidase called peroxinectin, which presents a novel mechanism for PG synthesis, was discovered in fruit flies and other insects (30).

Despite considerable research focusing on lipids in the context of insect immunity, our understanding of eicosanoids and other lipid molecules in the immune responses of *D. melanogaster* is limited. This study presents strong evidence suggesting that PUFAs stimulate immunity in *D. melanogaster* and further validates the presence of a lipid signaling pathway in insects.

## Materials and methods

### Fly stock/maintenance

Fly strains were cultivated on D2 glucose medium obtained from Archon Scientific in Durham, North Carolina. The flies were maintained at a temperature of 25°C with 50% humidity, following a 12-h light and 12-h dark cycle.

### Bacterial stock maintenance

The methods used in this study were adapted from previous research (31). *Streptococcus pneumoniae* was grown by shaking in glass vials with 5 ml tryptic soy (TS) broth (Difco Laboratories, MI, Catalog # 211825), catalase (Sigma-Aldrich, Catalog #C9322), and streptomycin (Fisher Scientific, Catalog # BP910-50) at 37°C with 5% CO_2_ for 16–18 h. The culture was diluted in catalase (100 μl) and TS broth to yield a final volume of 20 ml in a flask and incubated shaking until the *A*_600_–0.4 (about 1 h). The culture was then diluted again to a final volume of 50 ml, with 150 μl catalase and incubated until the *A*_600_ ∼ 0.2–0.4 (above 0.5 is no longer in log phase). 5% glycerol was added to the final culture and stored in 1 ml aliquots at −80°C. To use the aliquots, one tube was thawed, spun down at 14,000 rpm for 4.5 min, the supernatant was removed, and the pellet was resuspended in the desired amount of phosphate-buffered saline (PBS) [(160–180 μl yields ∼ 100,000 colony-forming unit (CFUs)] and serially diluted to yield the appropriate CFU doses. For quantification of CFUs, *S*.*p*. was plated on TS agar plates (Difco Laboratories, MI, Catalog # 236950) supplemented with 50 ml/L sheep’s blood (HemoStat Laboratories, Dixon, CA, Catalog # 50-863-755) and 200 mg/L streptomycin at 37°C with 5% CO_2_ for 16–18 h. *S. pnueumoniae* was heat-killed (HK) by incubating in a 56°C water bath for 30 min. The culture was then plated on TS agar plates to confirm the bacteria was HK. *Listeria monocytogenes* [serotype 4b, 19115, (ATCC, VA)] was also grown in batches in brain heart infusion (BHI) medium (Difco Laboratories, MI, Catalog # 299070) at 37°C in aerobic conditions. Cultures were grown overnight in a flask inoculated with a fresh colony and diluted again under log phase (below *A*_*600*_–0.2) and grown up to the desired *A*_600_ (∼0.4). The entire volume was transferred to a 50 ml centrifuge tube for vortexing. Before freezing, a 5% glycerol solution was added to the culture, and 1 ml aliquots were stored at −80°C. To use the aliquots, one tube was thawed, spun down at 14,000 rpm for 5 min, the supernatant was removed, and the pellet was resuspended in the desired amount of PBS (90–100 μl yields ∼ 100,000 CFUs) and serially diluted to yield the appropriate CFU doses. For quantification of CFUs, *L*.*m*. was plated on BHI agar plates at 37°C for 16–18 h. *L. monocytogenes* was HK by incubating in a 72°C water bath for 30 min. The culture was then plated on BHI agar plates to confirm the bacteria was HK.

### Fly lipid coinjections and survival assay

The methods employed in this study were adapted from previous research (31). Male flies aged 5–7 days were used for injections and metabolomics assays. The flies were anesthetized using CO_2_ and injected with various CFU doses of *S. pneumoniae*. The injections were performed with precise control, delivering a total volume of 50 nl, using a MINJ-FLY high-speed pneumatic injector (Tritech Research, CA) and individually pulled glass needles calibrated for accuracy. To examine the effects of fatty acids, different lipids including LA, AA, alpha-linolenic acid, oleic acid, and all PGs (obtained from Cayman Chemical, Ann Arbor) were dissolved in ethanol. Prior to injection, the lipids were freshly diluted in PBS for coinjection purposes. Injection sites were targeted close to the junction of the abdomen and thorax, slightly ventral from the dorsal–ventral cuticle axis, which could be easily observed beneath the haltere. For survival studies, one biological replicate is 60 flies, and experiments were carried out with three biological replicates per treatment. The experiments were replicated at least three times, involving a minimum of 180 flies per treatment group. Flies were injected with the CFU dose or a control of PBS and then placed in groups of 30 per vial. Flies injected with the human pathogen *S. pneumoniae* were maintained at a temperature of 28°C with 50% humidity. Daily monitoring of fly mortality was conducted, and the number of deceased flies was recorded. Kaplan-Meier survival curves were generated using GraphPad Prism software, and statistical analysis was performed using log-rank analysis (Mantel-Cox).

### PO activity

Methods adapted from Parks *et al.* (31). Flies were injected with 10,000 CFUs of HK *L. monocytogenes* to elicit an immune-induced melanization cascade. PO activity was measured as previously described (31). To collect hemolymph, 50 flies 6 h postinjection were pricked through the thorax and placed in a pierced 0.5 μl Eppendorf tube. Samples were centrifuged at 10,000 *g* for 20 min at 4°C. Using a clear 96-well plate, each well contained 160 μl of levodopa (3 mg/ml) dissolved in phosphate buffer (37.5% 1 M potassium phosphate and 62.5% 1 M sodium phosphate, pH 6.5), 5 μl of hemolymph sample, and 5 μl CaCl2 (20 mM). PO activity was measured by kinetic reads at 29°C at 492 nm every minute for 180 min with 3 s of shaking between reads. The *A* of a blank control was subtracted from all biological values. One biological replicate is 50 flies, and experiments were carried out with three biological replicates per treatment. Data were plotted as mean + standard error of the mean by taking the peak *A* value (timepoint ∼ 60 min). Statistics shown as ordinary one-way ANOVA with Dunnett’s multiple comparisons test.

### AMP gene expression—qPCR

Total RNA of 15 *S. pneumonia*-infected flies were extracted 24 h postinjection using TRIzol® reagent (Invitrogen, Carlsbad, CA) according to manufacturer instructions. Concentration and purity of RNA was determined by NanoDrop (Thermo Scientific™ NanoDrop™ 2000c, Catalog # ND-2000C). Total RNA was extracted and reverse-transcribed into cDNA using the Superscript IV™ Reverse Transcriptase (Thermo Fisher Scientific, Carlsbad, CA, Catalog # 18090010) following the manufacturer's protocol, in a MultiGene OptiMax Thermal Cycler (Labnet international, NJ). 2.5 μM of Oligo(dT)20 was used as reverse transcription primers. qPCR was performed using the PerfeCTa SYBR-Green FastMix (VWR—Quanta BioSciences, MD, Catalog # 95072-250) in a final volume of 10uL for each qPCR reaction. Gene-specific primers for Defensin, Drosomycin, Diptericin, Metchnikowin, and the housekeeping gene Tubulin (Integrated DNA Technologies, IA) were used, and all primers were added to a final concentration of 400 nM. Primer sequences are listed in supplemental information (supplemental Table S4). One biological replicate is 15 flies, and experiments were carried out with three biological replicates per treatment. All the samples were run in a minimum of three technical replicates in a thermocycler (CFX96 Touch™ Real-Time PCR Detection Systems, Hercules, CA) with the following steps: 95°C for 30 min, followed by 40 cycles of 95°C for 15 s, 55°C for 30 s, and 68°C for 30 s followed by a melt curve to check for off target amplification. Relative normalized expression was calculated by the ΔΔCq method using the housekeeping gene (Tubulin). Plots shown as bar graphs with individual points representing each biological replicate. Statistics shown as two-way ANOVA with Dunnett's multiple comparisons test done in GraphPad Prism.

### pHrodo phagocytosis

Phagocytic activity was measured using the pHrodo Red *E. coli* BioParticles Conjugate for Phagocytosis (Thermo Fisher Scientific, Catalog #P3536) and pHrodo Green *S. aureus* BioParticles Conjugate for Phagocytosis (Thermo Fisher Scientific, Catalog #P35382). The pHrodo Red *E. coli* and pHrodo green *S. aureus* were each resuspended in PBS to a working concentration of 4 mg/ml and then diluted 1:4 in PBS for injection. Flies were injected with pHrodo Red *E. coli* (1 mg/ml) in PBS or pHrodo Red *E. coli* (1 mg/ml) plus 250 μM LA, AA, or PGE2. Flies were injected with the pHrodo green *S. aureus* (1 mg/ml) in PBS or pHrodo green *S. aureus* (1 mg/ml) plus 250 μM LA, AA, or PGE2. Injected flies were incubated at 28°C with 50% CO_2_ for 1 h. To prepare for imaging, flies were placed in −80°C for 20 min and then fly wings were removed using microdissecting scissors and placed on ice for subsequent imaging. The dorsal side of the abdomen was imaged with an X-Cite® 120Q fluorescence lamp, and a ZEISS Axiocom 506 Color microscope camera attached to a ZEISS SteREO Discovery V12 microscope at 10× magnification. One biological replicate is three flies, and experiments were carried out with three biological replicates per treatment. ImageJ software was used to measure area-normalized total fluorescence of isolated red channels. Prior to analysis, images were preprocessed and denoised (median filter, 2-pixel radius) and background-subtracted (rolling ball subtraction, 50-pixel radius). Thresholding was used for segmentation to refine the objects using intensity-threshold algorithm in FIJI (Shanbhag, 30, 255). After processing, the areas containing fluorescent signals from phagocytosis were measured and area-normalized and reported as relative fluorescence. Statistics were shown as one-way ANOVA with Dunnett’s multiple comparisons test, with error bars depicting mean with standard error of the mean.

### Hemolymph collection for mass spectrometry

LA (Cayman Chemical, Ann Arbor) and AA (Cayman Chemical, Ann Arbor) were freshly diluted in PBS to a working concentration before injection. Two hundred flies were grouped per replicate, and five replicates were tested per treatment. For fatty acid analysis, five groups of 200 flies each were injected with either PBS or 1 mM LA (2,000 flies total). For eicosanoid analysis, five groups of 200 flies each were injected with either PBS or 1 mM AA (2,000 flies total). For analysis of lipids after bacterial infection, five groups of 200 flies each were injected with PBS or 7,000 CFU of *S. pneumoniae* (2,000 flies total). For hemolymph collection, groups of 200 flies were anesthetized with CO_2_ and were pierced through the thorax with a clean tungsten needle. Flies were placed in a pierced 0.5 ml Eppendorf tube within a 1.5 ml Eppendorf tube and centrifuged for two rounds of 10 min at 10,000 rpm at 4°C with a gentle mixing in between rounds. The supernatant of the collected hemolymph was centrifuged for 10 min at 14,000 rpm to remove cells and debris. The supernatant was flash-frozen in liquid nitrogen and stored at −80°C until prepped for metabolomics experiments.

### Lipidomic analysis

Lipidomic analysis of oxylipins and free-fatty acid composition in fly hemolymph during infection with *S. pneumoniae* was performed by Lipotype GmbH, Dresden, Germany (https://www.lipotype.com/). The following sample preparation steps were performed. A total of 10 μl of hemolymph was mixed with a combination of antioxidants and an internal standard comprising of 14,15-DHET-D11, 15-HETE-d8, 20-HETE-d6, 8,9-EET-d11, 9,10-DiHOME-d4, d4-12(13)-EpOME, d4-13-HODE, d4-PGB2, d4-PGE2-13,14-dihydro-15-keto, d4-PGF2a, LTB4-D4, and PGE2-D4, with a concentration of 1 ng each (obtained from Cayman Chemical, Ann Arbor). To induce protein precipitation and alkaline hydrolysis, methanol and sodium hydroxide were added to the sample, which was then incubated at 60°C for 30 min. After centrifugation and pH adjustment, the resulting supernatant was applied to Bond Elute Certify II columns (Agilent Technologies) for solid phase extraction. The eluate from the columns was evaporated using a heating block at 40°C under a stream of nitrogen, resulting in a solid residue. This residue was dissolved in 100 μl of methanol/water. Subsequently, the samples were subjected to liquid chromatography-tandem mass spectrometry analysis. An Agilent 1290 HPLC system equipped with a binary pump, multisampler, and column thermostat was utilized, employing a Zorbax Eclipse plus C-18 column with dimensions of 2.1 × 150 mm and a particle size of 1.8 μm. A gradient solvent system consisting of aqueous acetic acid (0.05%) and acetonitrile/methanol (50:50, v/v) was used. The flow rate was set at 0.3 ml/min, and the injection volume was 20 μl. The HPLC system was coupled with an Agilent 6,495 Triplequad mass spectrometer (Agilent Technologies, Santa Clara) equipped with an electrospray ionization source. Multiple reaction monitoring was employed in negative mode, with each compound monitored using at least two mass transitions. To assess data quality, the dynamic range of the instrument was established prior to the analysis. Based on this information, the limits of quantification and coefficients of variation for different lipid classes were determined. The limits of quantification generally ranged in the lower picogram range, varying depending on the specific analyte. The average coefficient of variation for a complete set of analytes was found to be less than 15%.

Lipidomic analysis of free fatty acids in fly hemolymph was performed at the University of California, San Diego, Lipidomics Core. Free fatty acids were extracted and analyzed by gas chromatography mass spectrometry essentially as described previously (32). A mix of deuterated fatty acids were spiked into 5 uL of hemolymph. Samples were extracted using methanol and isooctane, derivatized using PFBB, and analyzed by gas chromatography mass spectrometry on an Agilent 6890N gas chromatograph equipped with an Agilent 7683 autosampler (Agilent, Santa Clara, CA). Fatty acids were separated using a 15m ZB-1 column (Phenomenex) and monitored using single-ion monitoring identification. Analysis was performed using MassHunter software (Agilent, Santa Clara, CA). A 5 to 1 signal-noise ratio for the lower limit of quantification and a 3 to 1 signal-noise ratio for the limit of detection were used.

Lipidomic analysis of eicosanoids in fly hemolymph was performed at the University of California, San Diego, Lipidomics Core. A mix of 26 deuterated internal standards was added to 10uL of hemolymph. Eicosanoids were extracted by solid-phase extraction using Phenomenex Strata-X polymeric reversed phase columns as described (33). Samples were brought to dryness and taken up in buffer A (water/acetonitrile/acetic acid 60/40/0.02, v/v/v). Samples were analyzed using a Waters Acquity UPLC interfaced with an AB Sciex 6500 QTrap instrument. Chromatographic separation was achieved by a step gradient starting with 100% buffer A to 100% buffer B (acetonitrile/isopropanol 50/50, v/v) over 5 min. Standard curves were obtained in parallel using identical conditions. Data analysis was performed with Analyst and MultiQuant software packages (Applied Biosystems). We monitored 159 multiple reaction monitorings. A 5 to 1 signal-noise ratio for the lower limit of quantification and a 3 to 1 signal-noise ratio for the limit of detection were used.

## Statistics

All statistics were done with GraphPad Prism 9.1.0 for Mac. Statistical significance indicated with asterisks indicating the following *P* value cutoffs: *∗P < 0.05, ∗∗P < 0.01, ∗∗∗P < 0.001, ∗∗∗∗P < 0.0001*.

## Results

### *D. melanogaster* has endogenous long-chain PUFAs and can synthesize PGs when supplemented with AA

**Fig. 1:**
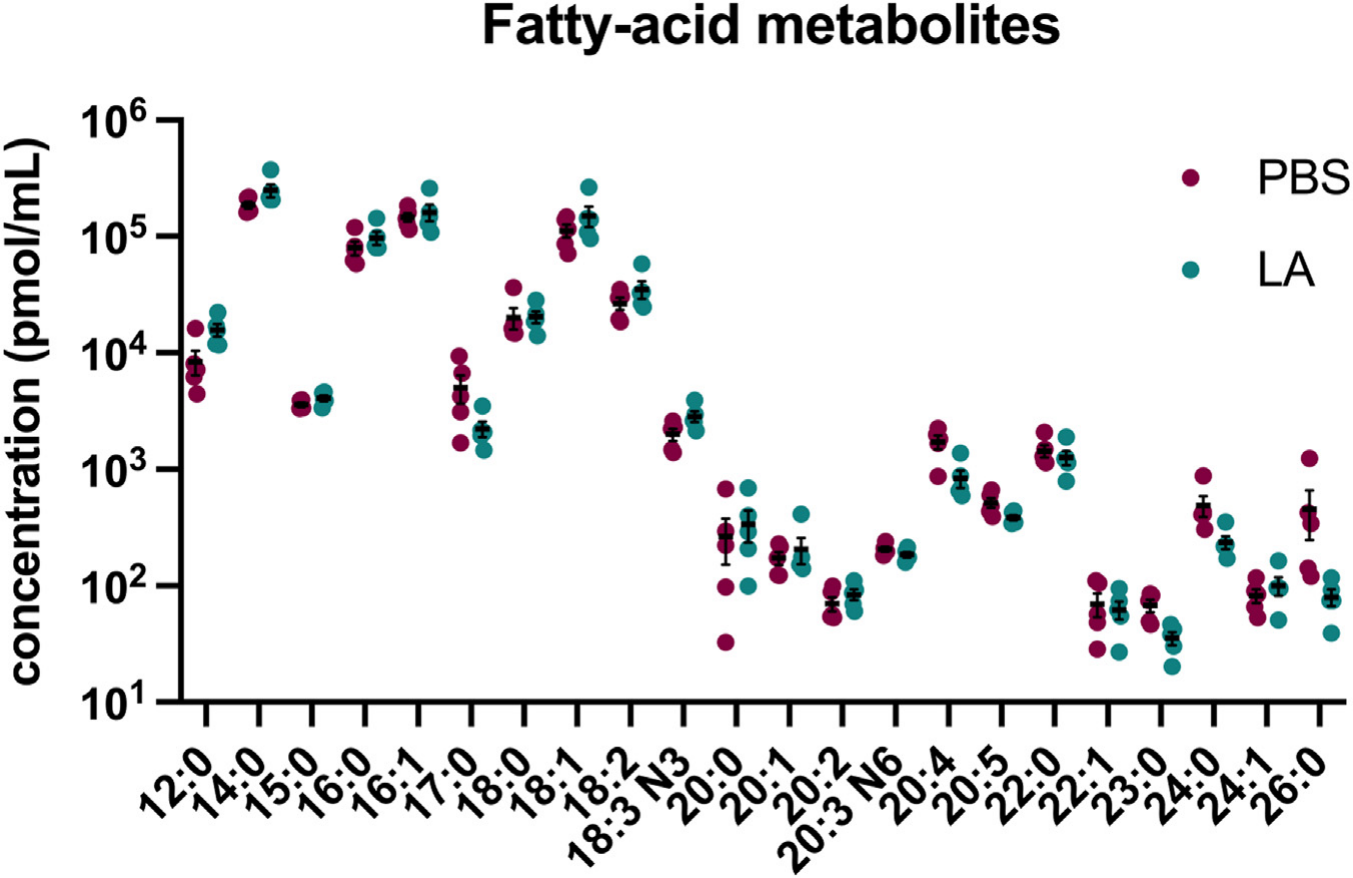
*Drosophila melanogaster* hemolymph contains long-chain and very-long-chain PUFAs. The lipids in the fly hemolymph include long-chain and very-long-chain fatty acids including C20, C22, C23, C24, and C26. Quantitatively, *D. melanogaster* contains higher concentrations of C14, C15, C16, and C18 PUFAs. Flies were injected with 1 mM linoleic acid (LA), and hemolymph samples were collected for mass spectrometry analysis at 6 h post injection. Control flies are flies injected with PBS. The experiments were repeated five times, with each treatment group consisting of 200 flies per replicate, totaling 2,000 flies: 1,000 per treatment. Data shown as bar graphs with individual points representing groups of 200 flies. The error bars represent the mean + SEM (standard error of the mean), and statistical analysis was performed with multiple unpaired t-tests. Abbreviations are as follows: 12:0 (lauric Acid), 14:0 (myristic acid), 15:0 (pentadecylic acid), 16:0 (palmitic acid), 16:1 (cis-9-palmitoleic acid), 17:0 (margaric acid), 18:0 (stearic acid), 18:1 (oleic acid), 18:2 (linoleic acid), 18:3 N3 (alpha-linolenic acid), 20:0 (arachidic acid), 20:1 (cis-gadoleic acid), 20:2 (11,14-eicosadienoic acid), 20:3 N6 (bishomo-gamma-linolenic acid), 20:4 (arachidonic acid), 20:5 (eicosapentaenoic acid), 22:0 (behenic acid), 22:1 (cis-erucic acid), 23:0 (tricosylic acid), 24:0 (lignoceric acid) 24:1 (nervonic acid), 26:0 (cerotic acid).

**Fig. 2:**
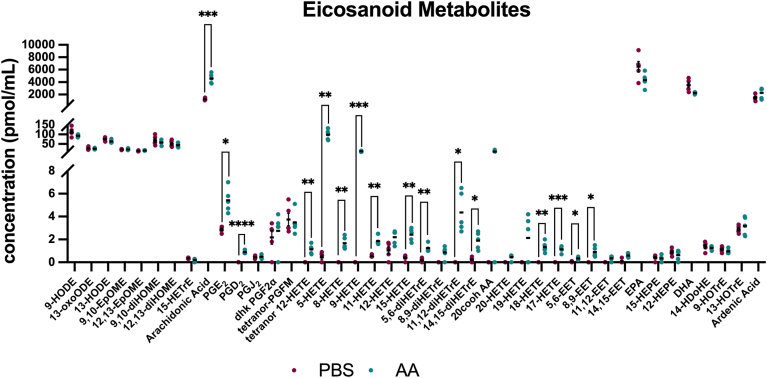
*D. melanogaster* can synthesize PGE_2_ and PGD_2_ when given arachidonic acid. Flies were injected with 1 mM arachidonic acid (AA), and hemolymph samples were collected for mass spectrometry analysis at 6 h postinjection. The eicosanoids PGE_2_, PGD_2_, tetranor 12-, 5-, 8-, 9-, 11-, 15-, 17-, and 18-HETE showed significant increase after AA treatment. Other eicosanoids such as 5,6-, 8,9-, 11,12-, and 14,15-diHETrE, along with 5,6- and 8,9-EET were also significantly increased. The experiments were repeated five times, with each treatment group consisting of 200 flies per replicate, totaling 2,000 flies: 1,000 per treatment. Data shown as bar graphs with error bars representing the mean + SEM (standard error of the mean), and statistical analysis was performed with multiple unpaired *t*-tests. Asterisks indicate the following *P* value cutoffs: ∗*P* < 0.05, ∗∗*P* < 0.01, ∗∗∗*P* < 0.001, ∗∗∗∗*P* < 0.0001.

To investigate if *D. melanogaster* was capable of endogenously producing AA from LA, a total of 2,000 individuals were injected with either PBS or 1 mM LA, and fly hemolymph was collected 6 h postinjection. The fatty acid composition of hemolymph samples was analyzed using gas chromatography-mass spectrometry. Treatment with LA did not significantly increase the concentration, measured in pmol/ml, of endogenous AA in the hemolymph or of any fatty acid measured (Fig. 1). However, this analysis further validated the presence of endogenous long-chain and very-long-chain fatty acids namely C20, C22, C23, C24, and C26 PUFAs, in detectable quantities in *D. melanogaster* (12) (Fig. 1). Fly hemolymph had higher concentrations of long-chain fatty acids with less than 20 carbons, with myristic acid (14:0) being the most abundant fatty acid detected (Fig. 1). In addition to identifying endogenous AA production, we tested if treatment with AA would stimulate eicosanoid production. A total of 2,000 individuals were injected with either PBS or 1 mM AA, and fly hemolymph was collected 6 h postinjection. The eicosanoid composition of the hemolymph samples was analyzed using liquid chromatography-mass spectrometry. For flies treated with AA, the COX-derived eicosanoids PGE_2_, PGD_2_ and LOX-derived eicosanoids 12-, 5-, 8-, 9-, 11-, 15-, 17- and 18-HETE displayed a significant increase in quantity (Fig. 2). Other eicosanoids such as 5,6-, 8,9-, 11,12-, and 14,15-diHETrE, along with 5,6- and 8,9-EET were also significantly increased in flies treated with AA compared to PBS injected flies (Fig. 2). This result highlights that *D. melanogaster* can produce AA-derived COX, LOX, and CYP450 products (7). These results strongly support the presence of lipid signaling pathways in insects. All fatty acids and eicosanoids screened in Fig. 1 and 2, respectively, are reported in the supplementary (supplemental Tables S2 and S3).

### LA induces phagocytosis of *Staphylococcus aureus*

**Fig. 3:**
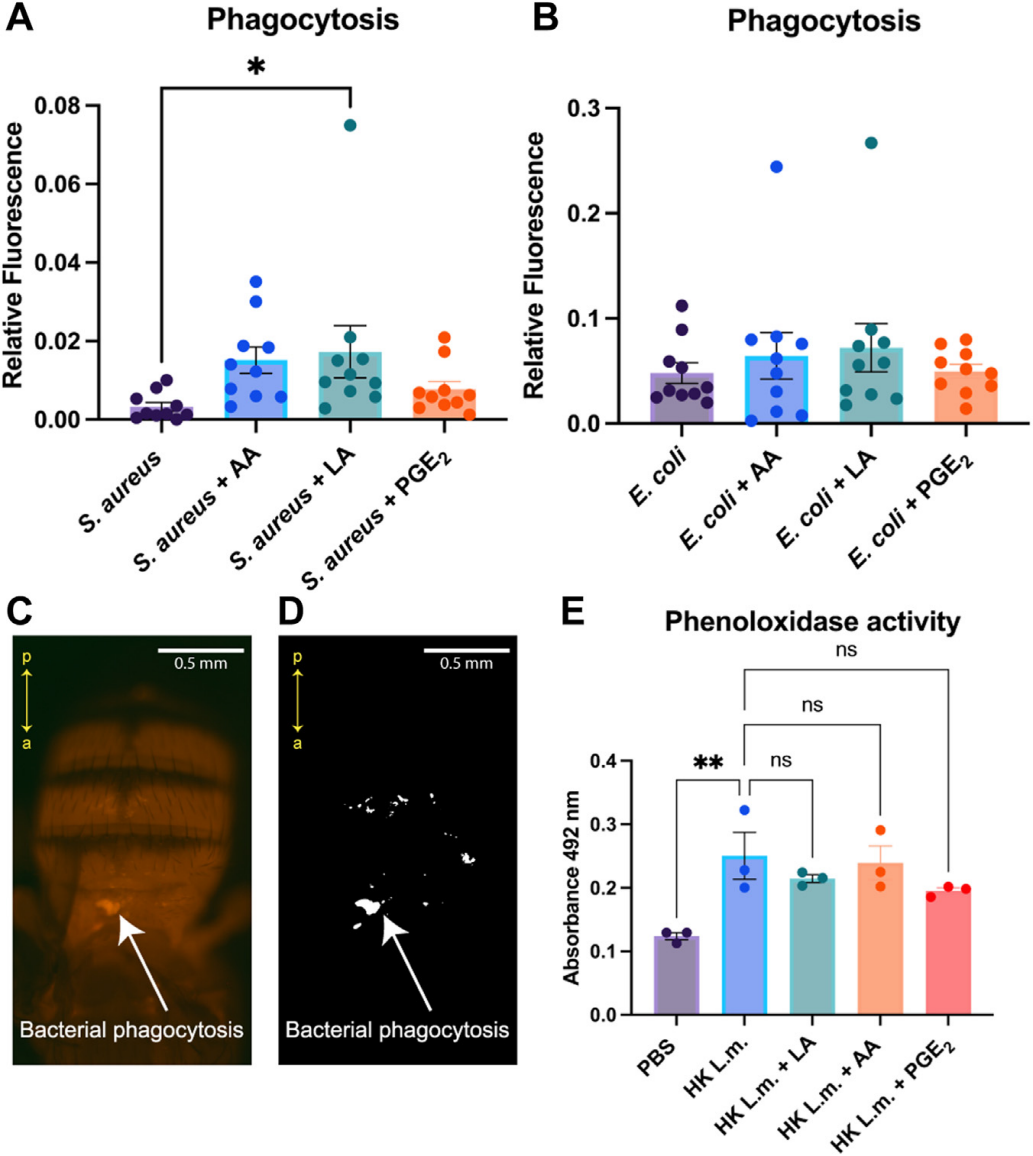
Phagocytosis of *Staphylococcus aureus* is increased by linoleic acid. (A) Phagocytosis of gram-positive bacteria *S. aureus* was measured with the pHrodo assay. Injection with 250 μM linoleic acid (LA) plus *S. aureus* significantly increased phagocytosis compared to bacteria alone. Injection with 250 μM arachidonic acid (AA) or prostaglandin E_2_ (PGE_2_) did not affect phagocytosis of *S. aureus*. Experiments were biologically triplicated with at least three flies per biological replicate. Data shown as bar graphs with individual points representing all experimented flies. Error bars depict mean with SEM (standard error of the mean). Statistics displayed as ordinary one-way ANOVA with Dunnett’s multiple comparisons test. (B) Phagocytosis of gram-negative bacteria *E. coli* was unaffected by the addition of 250 μM LA, AA, or PGE_2_. Experiments were biologically triplicated with at least three flies per biological replicate. Data shown as bar graphs with individual points representing all experimented flies. Error bars depict mean with SEM. Statistics displayed as ordinary one-way ANOVA with Dunnett’s multiple comparisons test. (C) After injection, phagocytosis of fluorescently labeled bacteria can be seen on the dorsal side of the upper abdomen using the pHrodo assay. Image depicts fluorescence signal 1 h postinjection with *S. aureus* and 250 μM LA. White arrows show bacterial phagocytosis and (a)-anterior and (p)-posterior axes (yellow) indicate the orientation. (D) Images were processed in ImageJ to isolate fluorescent areas. Image shows postprocessed image of injection with *S. aureus* and 250 μM LA. White arrows show bacterial phagocytosis and (a)-anterior and (p)-posterior axes (yellow) indicate the orientation. (E) Phenoloxidase activity was measured 6 h postinjection with either PBS control, 10,000 cells heat-killed *Listeria monocytogenes* (HK *L.m.*), a known melanizer, or *L.m.* plus 250 μM LA, AA, or PGE_2_. No significant change in PO activity was observed in lipid plus bacteria-injected groups. Phenoloxidase was significantly increased in flies injected with heat-killed *L m* compared to PBS injection alone. Experiments were biologically triplicated with 50 flies per biological replicate. Data shown as bar graphs with individual points representing biological replicates. Statistics displayed as ordinary one-way ANOVA with Dunnett’s multiple comparisons test. Error bars depict mean with SEM (standard error of the mean). Asterisks indicate the following *P* value cutoffs: *∗P < 0.05, ∗∗P < 0.01*.

To delineate how PUFAs potentially stimulate immunity, we assessed various indicators of immune response, such as phagocytosis, PO activity, and AMP expression. Phagocytosis is a crucial cellular immune process in insects and was quantified in *D. melanogaster* through the injection of fluorescently labeled conjugates of *E. coli* and *S. aureus* (34, 35). These conjugates fluoresce upon exposure to the low pH environment of the lysozyme, and the total fluorescent area can be quantified. To measure if lipids affect phagocytosis in vivo, we injected these bacterial conjugates with either 250 μM LA, AA, or PGE_2_. Our findings demonstrated that only LA significantly enhanced phagocytosis activity of *S. aureus* 1-hour postinjection as indicated by an increase in relative fluorescence compared to *S. aureus* alone (Fig. 3A). No changes in relative fluorescence were observed during experiments with *E. coli*, suggesting that at a 250 μM concentration, LA, AA, or PGE_2_ did not affect phagocytosis of *E. coli* (Fig. 3B). A visual indication of phagocytized *S. aureus* appearing as fluorescence on the dorsal side of the upper abdomen of the fly, along with the respective postprocessed image for analysis, are shown in Fig. 3C, D. In addition to phagocytosis, we evaluated changes in PO activity in hemolymph using the DOPA assay. In this assay, PO catalyzes the conversion of levodopa (L-DOPA) substrate into a pigment called dopachrome (orange to red), whose absorbance can be measured at 492 nm (36). Flies were injected with 10,000 CFUs of HK *Listeria monocytogenes,* and hemolymph was collected 6 h later for PO quantification. We used HK *L. monocytogenes* as a positive control to ensure our assay resulted in an increase in PO activity as it is a bacterium known to trigger robust melanization (27, 37). Indeed, injection with HK *L. monocytogenes* significantly increased PO activity compared to PBS alone (Fig. 3E). To measure if PUFAs and PGE_2_ affect PO activity, we coinjected HK *L. monocytogenes* with 250 μM LA, AA, or PGE_2_ and collected hemolymph 6 h postinjection. Our results showed that treatment with either 250 μM LA, AA, or PGE_2_ did not result in significant changes in PO activity compared to HK *L. monocytogenes* alone (Fig. 3E). These results indicate that at a 250 μM concentration, LA, AA, or PGE_2_ have no effect on PO activity.

### LA and AA increase expression of AMP genes in the Toll and Imd pathway

**Fig. 4:**
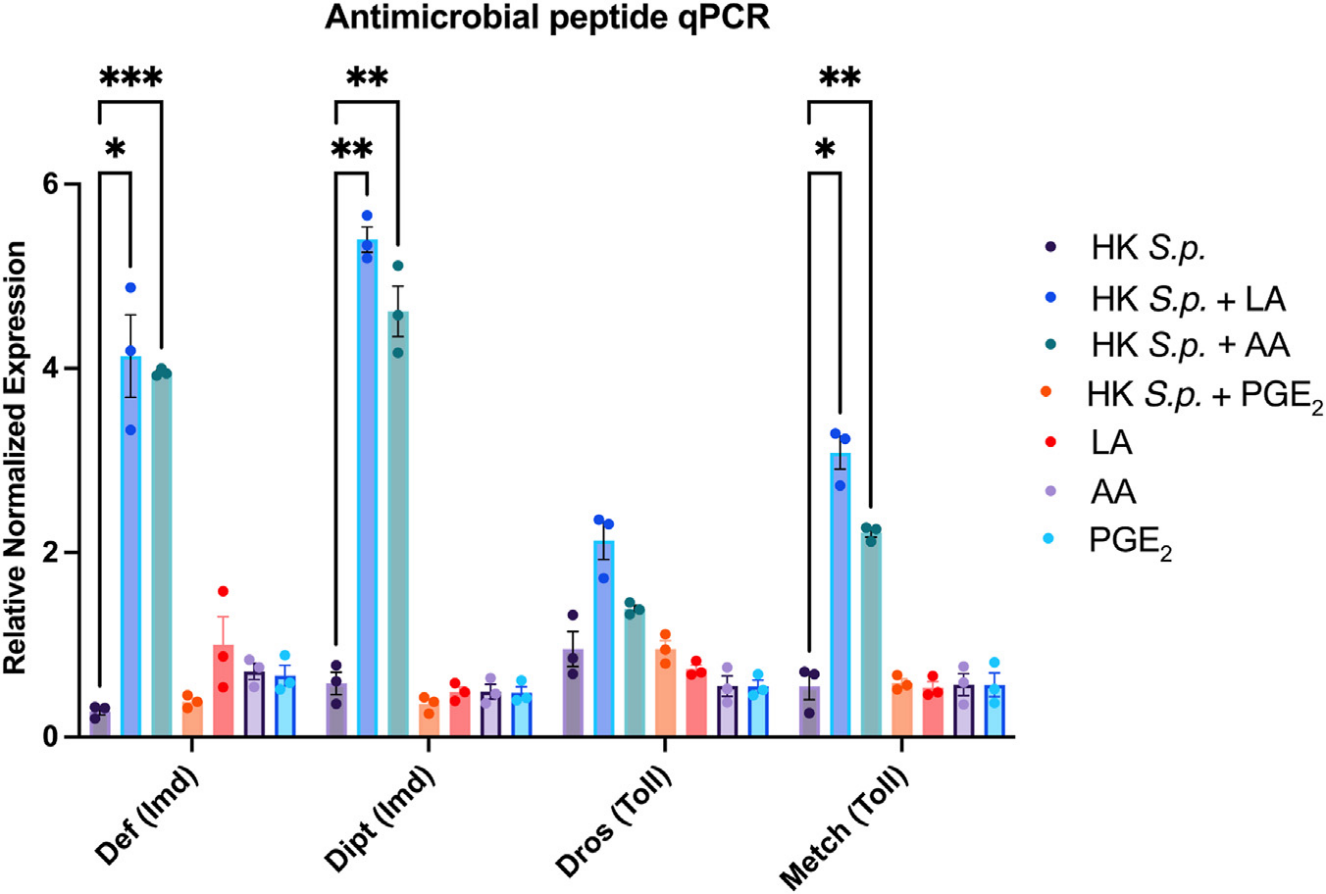
Linoleic acid and arachidonic acid upregulate Imd and Toll pathway dependent AMP expression only at immune-stimulated conditions. Antimicrobial peptide (AMP) expression was measured with qPCR 24 h after injection with heat-killed *S. pneumoniae* (HK *S.p.*), heat-killed *S.p.* plus 250 μM linoleic acid (LA), arachidonic acid (AA), or prostaglandin E_2_ (PGE_2_), or lipid alone. Four different AMPs from the Imd or Toll pathway were measured*, defensin* (Imd), *diptericin* (Imd), *drosomycin* (Toll), and *metchnikowin* (Toll). *Defensin, diptericin,* and *metchnikowin* were all significantly upregulated after injection with heat-killed *S.p*. and 250 μM LA or AA compared to heat-killed *S.p.* alone. Lipids alone showed no change in AMP expression relative to heat-killed *S.p.* alone. Experiments were biologically triplicated with 15 flies per biological replicate. Data shown as bar graphs with individual points representing biological replicates. Error bars depict mean with SEM (standard error of the mean). Statistics shown as two-way ANOVA with Dunnett's multiple comparisons test. Asterisks indicate the following *P* value cutoffs: *∗P < 0.05, ∗∗P < 0.01, ∗∗∗P < 0.001.*

To evaluate the role of lipids in humoral immunity, we measured expression of Toll and Imd-dependent AMPs. Fly treatment groups were injected with HK *Streptococcus pneumoniae* (*S.p.*) and HK *S.p* plus 250 μM LA, AA, or PGE_2_. Injections with 250 μM lipids alone were tested as controls. The expression of two Imd-dependent AMPs, *defensin* and *diptericin*, and two Toll-dependent AMPs, *drosomycin* and *metchnikowin,* was measured. AMP expression 24 h postinjection was quantified using qPCR. Primer sequences are included in the supplementary (supplemental Table S3). Treatments with 250 μM LA or 250 μM AA resulted in significant upregulation of endogenous *defensin*, *diptericin*, and *metchnikowin* in the presence of HK *S.p* (Fig. 4). Flies coinjected with 250 μM PGE_2_ and HK *S.p.* showed no significant change to AMP expression compared to HK *S.p.* alone. Furthermore, treatment with just lipids did not change endogenous AMP expression compared to HK *S.p* indicating that lipids alone do not enhance AMP expression (Fig. 4). These results indicate that LA and AA have a stimulatory effect on the Toll and Imd pathway only in immune-stimulated conditions (38, 39).

### PUFAs display bactericidal effects on *Streptococcus pneumonia*

We attempted to demonstrate a phenotypic stimulatory effect on immunity by coinjecting flies with 7,000 cells of *S.p.* and various concentrations of PUFAs and PGs. Each PUFA and PG tested increased survival in the fly at specific concentrations compared to *S.p.* control (supplemental Figs. S2 and S3). An increase in survival could be seen at different minimal concentrations depending on the PUFA and PG. Oleic acid, LA, AA, and ALA increased survival at initial concentrations of 100, 250, 50, and 50 μM, respectively (supplemental Fig. S2). PGs F2_α_ and F2_β_ increased survival at an initial concentration of 100 μM, while PGs D2 and E2 were 250 μM and 50 μM respectively (supplemental Fig. S3). To determine whether the increase in survival impacted microbial load, we attempted to quantify bacterial CFU for *S.p.* at 0 h and 24 h after coinjection with the PUFAs and PGs. The plating experiments of *S.p.* plus LA or *S.p.* plus AA showed no growth at 0 and 24 h, indicating the PUFAs and PGs are bactericidal to *S.p.* (supplemental Fig. S4). Therefore, the survival data could not delineate if the immunostimulatory effects or bactericidal effects of the lipids increased survival against *S.p.* infection.

## Discussion

The innate immune response in *Drosophila* involves two pathways: the humoral immune response, which produces AMPs, and the cellular immune response, which involves hemocyte cells controlling pathogen growth in circulation (23). The immune response mechanisms in *Drosophila* and mammals are highly conserved (24). Previous research indicates that bacterial infections increase phospholipid synthesis in the fat body, the main organ for humoral immune response in *Drosophila*, which can be converted to eicosanoids (40).

We demonstrated that the lipid profile of *D. melanogaster* includes long-chain fatty acids necessary for eicosanoid production (Fig. 1). This finding further validates our previous research that also detected endogenous C20 and C22 lipids in the fly (12). We examined the food source given to the flies to ensure that long-chain fatty acids were not being introduced in their food source. Mass spectrometry analysis of the fly food showed no C20 or C22 lipids, providing more evidence that long-chain fatty acids in the fly are being endogenously produced (data not shown). While we confirmed the presence of C20 and C22 eicosanoid precursors, we were not able to show the increase of AA when the fly is treated with LA. It is possible that conversion of LA to AA is done in the presence of a pathogen, as both LA and AA showed an increase in AMP expression in the presence of HK *S. pneumoniae* (Fig. 4). Future experiments, which examine fly hemolymph after LA treatment in the presence of a bacterial challenge, could potentially show direct increase of AA or other C20 lipids. Furthermore, it is possible that because detection of the lipids from the lipid panel was done 6 h postinjection, the excess LA was converted to other C18 oxylipins which were not screened in the lipid panel. A future lipidomics experiment, including an oxylipin panel, measured at an earlier time point would be needed to test this hypothesis. The concentration of lipids in the fly hemolymph as shown in our lipid profile analysis (Figs. 1 and 2) was measured based on pooled hemolymph of 200 flies per replicate. Fly hemolymph volume and thus PUFA concentration will have some variance among each individual fly. Our experiments controlled the dose given to each fly, and an amount was given as close to physiological relevance as we could control to generate a measurable response in our assays. Fly concentration of LA was around 10,000 pmol/ml which is 10 μM. Concentration for AA was around 1,000 pmol/ml which is 1 μM. Our experiments for determining changes in immune response used a concentration of LA that was 25 times more than what was determined in the hemolymph for LA and 250 times more for AA. It is important to note that our findings do not suggest the concentration amount of LA and AA we used as a treatment is required to generate an immunostimulatory effect in the fly. The findings do demonstrate that an overall increase of AA led to downstream eicosanoid production in the fly, and LA and AA stimulated certain immune responses. Future experimentation with a more physiologically relevant dose is needed for more physiologically relevant quantification.

The presence of C20 lipids is necessary for production of key eicosanoids such as PGs. PGs, presumably synthesized from AA by peroxinectin (or another COX-like enzyme) in *Drosophila*, play critical roles in development, reproduction, and immunity (30). The hypothesis that C20 lipids are converted to downstream immunomodulatory PGs in insects is further strengthened by the increased production of PGE_2_ and PGD_2_ in *D. melanogaster* after treatment with AA (Fig. 3). Interestingly, PGE_2_ has a proposed direct synthesis pathway in insects (41). Eicosanoid receptors are likely highly conserved, and the ligands capable of activating downstream immune pathways are diverse, although only a few are endogenously found in flies. When treated with AA, *D. melanogaster* was able to produce COX, LOX, and CYP450 eicosanoid products (Fig. 2). Particularly interesting is the increased production of 5-HETE which is converted to leukotriene A_4_ by 5-LOX (42, 43). This indicates that the ability to produce leukotrienes via the LOX pathway may be present in *D. melanogaster*. Recent studies have also shown that HETEs are able to be synthesized without enzymes by oxidative stress, highlighting a potential alternative method for synthesizing oxylipins (44). Genes for LOX-like enzymes in *D. melanogaster* have been identified via computational methods (45). Future experimentation, however, will be crucial for experimentally validating whether these genes are able to generate LOX products at the molecular level. CYP450 enzymes have been shown to be present in all tissues of insects, producing molecules that are involved in growth development, feeding, pesticide resistance, and plant toxin tolerance (46, 47). Our data show that *D. melanogaster* produces these CYP450-derived eicosanoids when given the lipid precursor AA (Fig. 2), providing further evidence of a lipid signaling pathway that generates eicosanoids in insects.

We evaluated the effects of LA, AA, and PGE_2_ on downstream immunity in the fly. Fly immunity starts with pathogen-specific recognition by the Toll and Imd pathways, which then leads to either a cellular immune response by specialized hemocytes or a humoral immune response via production of Toll- or Imd-specific AMPs secreted from the fat body (19, 26). Melanization is independent of the Toll and Imd pathways and is dependent on the proPO-PO cascade (22, 23). Our findings showed that all lipids had no effect on PO activity (Fig. 3E), but LA and AA caused an increase in the expression of the AMPs *metchnikowin, diptericin,* and *defensin*, but not *drosomycin* (Fig. 4). LA significantly increased phagocytosis against *S. aureus*, but not against *E. coli* (Fig. 3A, B). Previous studies in mammals have linked C18s to increased phagocytosis in alternatively activated macrophages, specifically by ALA (48). LA has also been shown to be a potent inducer of neutrophilic respiratory burst, a process that is involved with phagocytosis of neutrophils (49). More experimentation is needed to elucidate if more C18s, such as oxylipins produced endogenously in insects (supplemental Fig. S1), can stimulate phagocytosis if it is pathogen specific. An increase of AMP expression by LA and AA provides evidence of immunostimulation by lipid signaling mechanisms. In our previous research, a parasitic nematode sPLA_2_ was able to increase LA but generated reduced AMP expression (12). This effect however was due to reduction of hemocytes that was reported in our studies, leading to reduced synthesis of AMPs (50). PGE_2_ did not affect AMP expression or phagocytosis, but there are potentially multiple reasons for this. PGE_2_’s role in immunity specifically could be in another aspect of the cellular immune response such as encapsulation or nodulation. PGE_2_ has a short-half life, thus the probability for PGE_2_ to remain structurally viable during circulation after injection to exert an effect is low. It is also possible other synthesized eicosanoids from the COX pathway, such as PGD_2_, or from the LOX or CYP450 pathway, are responsible for stimulation of AMP expression specifically. Overall, these findings show that LA and AA both have immunostimulatory capabilities on downstream immune responses in insects.

It is important to note that we attempted to phenotypically demonstrate an increase in survival when introducing PUFAs and eicosanoids to the fly in the context of an infection. We saw increased survival at various doses of PUFAs and eicosanoids coinjected with *S.p.* compared to bacteria alone (supplemental Figs. S2 and S3). The PUFAs used in this study however are also bactericidal to gram-positive bacteria, and thus when attempting to plate S.p. plus LA or *S.p.* plus AA coinjections for CFUs, growth was not observed (supplemental Fig. S4) (21, 22). Therefore, the survival assays we utilized could not delineate between lipids acting on the bacteria directly or in the fly to stimulate immunity. Our findings reported in this study overall demonstrate immunostimulation of key immune response mechanisms in the fly from PUFAs. Future insect phenotypic studies will need to account for bactericidal properties of lipids when designing viable assays for assessing survival.

**Fig. 5:**
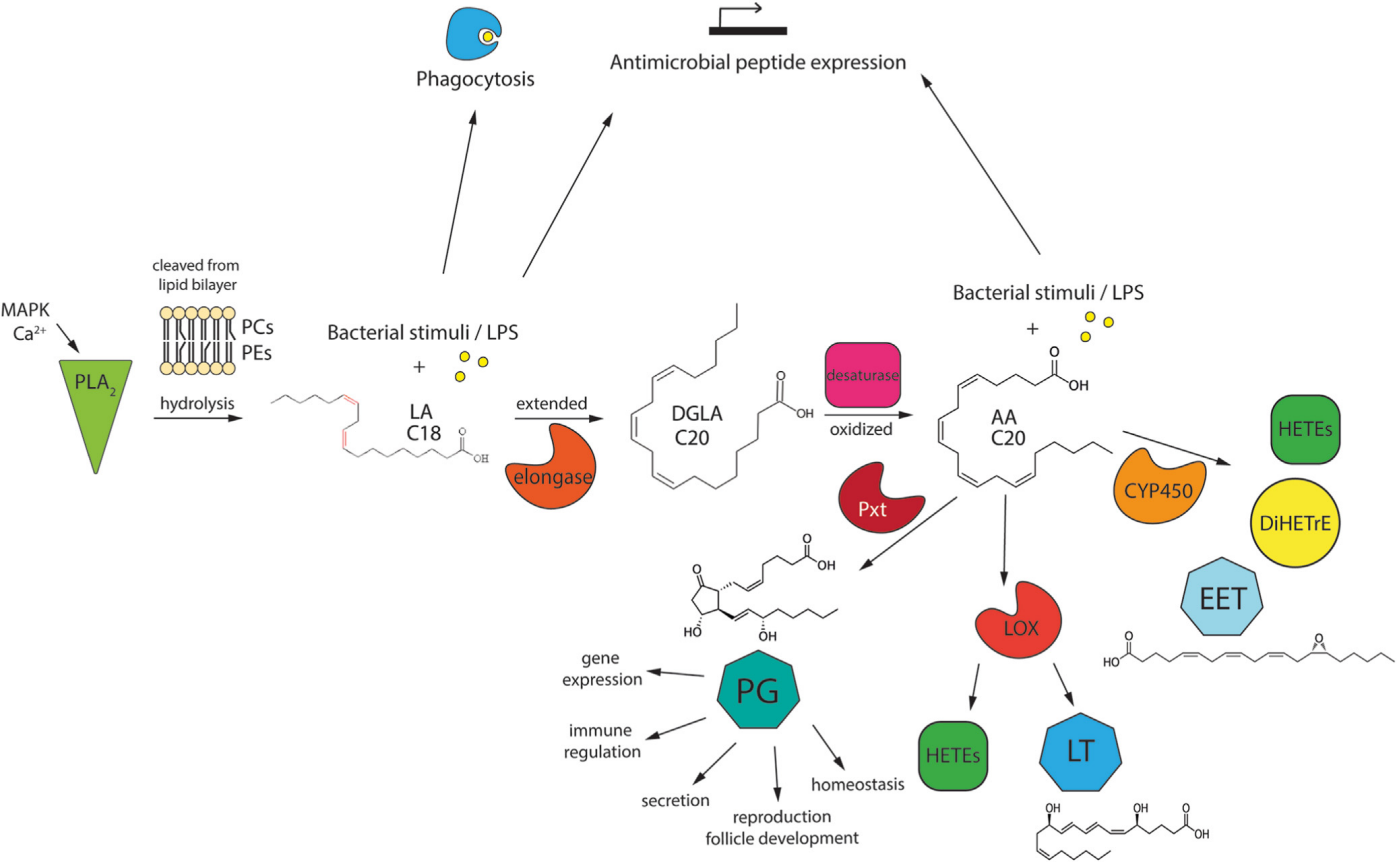
Diagram of the hypothesized lipid signaling pathway in *Drosophila melanogaster*. According to the proposed pathway lipid signaling linoleic acid (LA) is cleaved from the membrane by phospholipase A2 (PLA_2_) and synthesized to arachidonic acid (AA) via an elongase and desaturase. Dihomo gamma-linolenic acid (DGLA) is a 20-carbon intermediate product between LA conversion to AA. AA is a substrate for three different metabolic pathways leading to oxylipins. AA is oxygenated into leukotrienes (LT) and hydroxyeicosatetraenoic acids (HETEs) via lipoxygenase (LOX). AA can be converted into epoxyeicosatrienoic acid (EET), dihydroxy-eicosatrienoic acid (DiHETrE), and HETEs via cytochrome P450 (CYP450). AA is oxygenated and converted to prostaglandins (PG) via peroxidase enzyme (Pxt) which has been proposed to be an insect cyclooxygenase ortholog. PGs have been shown to play a role in gene expression, immunity, secretion, reproduction, and insect homeostasis. Current findings demonstrate that with the addition of bacterial stimuli, LA and AA have been found to affect immune pathways in *D. melanogaster* including phagocytosis and antimicrobial peptide expression.

In mammals, the eicosanoid pathway from AA branches into PGs, leukotrienes, and EETs, which communicate proinflammatory or anti-inflammatory signals (45). This study sheds light on a potential conserved system in insects where lipid signaling may also induce proimmune or antiimmune responses, depending on the activated pathways. We have constructed a figure that hypothesizes the potential lipid signaling pathway based on the findings of this study and previous research (Fig. 5). Overall, our findings demonstrate further evidence of a functional eicosanoid biosynthesis pathway in *Drosophila* and the role of lipid signaling in *Drosophila* immunity.

## Data availability

Raw data are available here: https://data.mendeley.com/preview/687br84fwp?a=c76d5da0-c7da-4f2f-a86a-94e6a976e6ef.

## Supplemental data

This article contains supplemental data.

## Conflict of interest

The author declares that they have no conflicts of interest with the contents of this article.
