# Supplementary material for: Polyunsaturated fatty acids stimulate immunity and eicosanoid production in Drosophila melanogaster
Source: J Lipid Res. 2024 Jul 26;65(9):100608. doi: 10.1016/j.jlr.2024.100608 (PMC11386307; doi:10.1016/j.jlr.2024.100608)
Supplement: Supplemental Information [file mmc1.pdf]

SUPPLEMENTAL INFORMATION:

Polyunsaturated fatty acids stimulate immunity and eicosanoid production in *Drosophila melanogaster*

Pakeeza Azizpor[1Δ], Ogadinma K. Okakpu[1Δ], Sophia C. Parks[1,2], Diego Chavez[1], Fayez Eyabi[1], Stephanie Martinez-Beltran[1], Susan Nguyen[1], and Adler R. Dillman[1*]

[1]Department of Nematology, University of California, Riverside, California, 92521, USA.

[2]Current affiliation, Department of Microbiology and Immunology, Stanford University, Stanford, California, 94305, USA.

[Δ] Equal contribution

*Corresponding: adlerd@ucr.edu

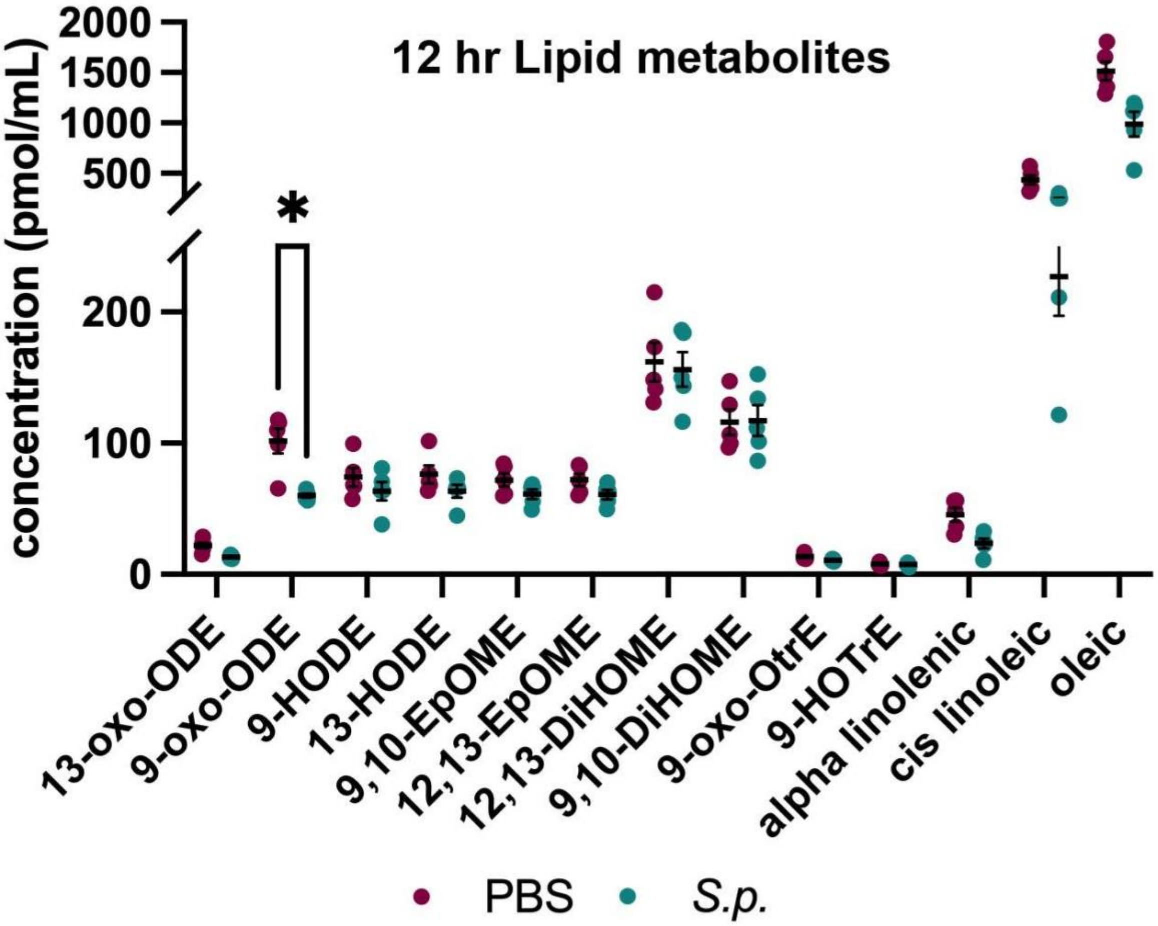

Supplemental Figure 1: A lipid metabolite of linoleic acid (LA) in fly hemolymph was significantly reduced 12 hours after a *Streptococcus pneumoniae* (*S.p.*) induced immune challenge. Flies were injected with 7,000 *S.p.* cells, and hemolymph samples were collected for mass spectrometry analysis at the 12-hour mark post-injection. Concentration of 9-oxo-ODE, a metabolite of linoleic acid metabolism via the lipoxygenase pathway, was significantly reduced in infected flies. The experiments consisted of 200 flies per replicate, five replicates were done per treatment, totaling 2,000 flies. Error bars represent the mean + SEM (standard error of the mean), and statistical analysis was performed using multiple unpaired t-tests. Asterisks indicate the following p-value cut offs: ** p<.05*.

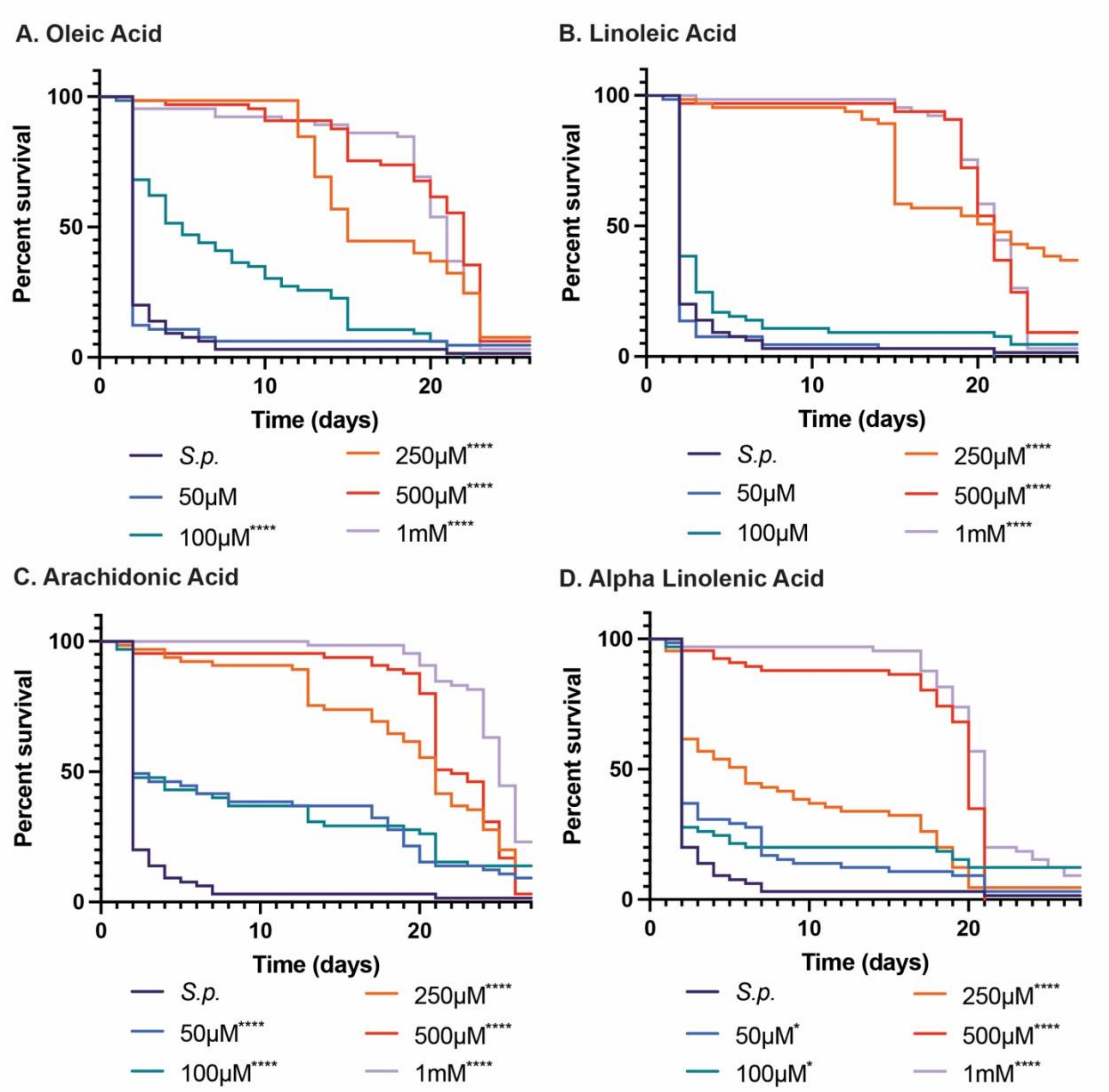

Supplemental Figure 2: In a dose-dependent manner, both upstream lipids and downstream oxylipins demonstrate the ability to rescue bacterial infections. Flies were injected with 7,000 *Streptococcus pneumoniae* (*S.p.*) cells along with various lipid doses. **A)** Oleic acid (OA) exhibits a significant beneficial effect on infection outcomes starting at 100 µM. **B)** Linoleic acid (LA) shows beneficial effects at 250 µM. **C&D)** Arachidonic acid (AA) and alpha-linolenic acid (ALA) are effective even at the lowest dose of 50 µM. Dark blue bars represent control groups injected with *S.p.* only. The experiments were replicated at least three times, with a total of at least 180 flies per treatment group. Statistical analysis was performed using the Log-rank test, and asterisks are used to indicate significant differences next to the experimental group labels below the graph.

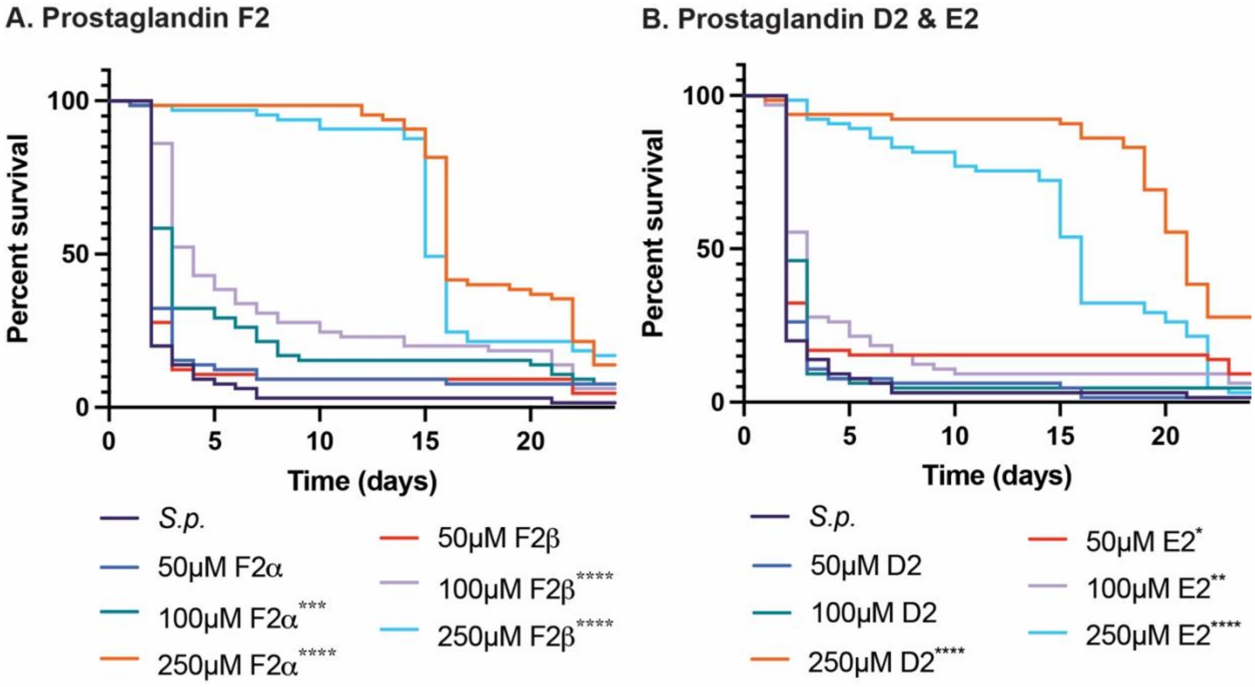

Supplemental Figure 3: Downstream prostaglandins have a significant positive impact on the outcome of bacterial infections. Flies were injected with 7,000 *Streptococcus pneumoniae* (*S.p.*) cells along with varying doses of prostaglandins. **A)** Prostaglandins $F2_{\alpha}$ and $F2_{\beta}$ demonstrate substantial improvement in infection outcomes at doses of 100 µM and 250 µM. **B)** Prostaglandin D_2 exhibits beneficial effects only at the highest dose of 250 µM, while prostaglandin E_2 shows a positive effect starting at a lower dose of 50 µM. The experiments were replicated at least three times, involving a minimum of 180 flies per treatment group. Statistical analysis was performed using the Log-rank test, and asterisks are used to indicate significant differences next to the experimental group labels below the graph.

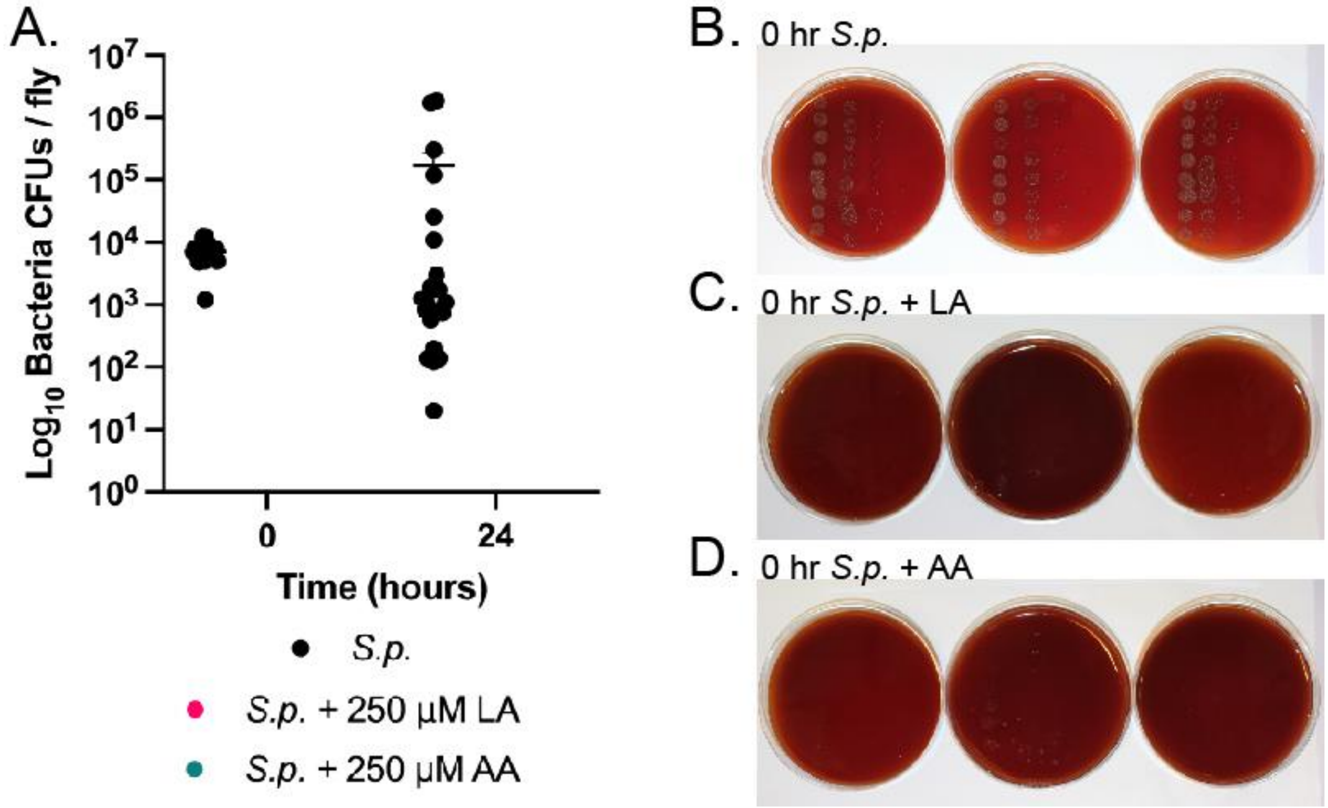

Supplemental Figure 4: CFU quantification of bacteria plus lipid co-injections. Flies were injected with ~ 7,000 *Streptococcus pneumoniae* (*S.p.*) cells alone or with either 250 µM linoleic acid (LA) or 250 µM arachidonic acid (AA). CFU quantification was measured at two time points; immediately after injection (0-hour) and 24 hours post injection. 24 flies per treatment group were sacrificed for bacterial dose quantification. **A)** No bacterial colonies were observed at either time point for *S.p.* plus 250 µM LA or *S.p.* plus 250 µM AA. All experiments were repeated at least three times. Data shown as a scatter plot, with individual points representing CFU per fly. Error bars depict mean with SEM (standard error of the mean). **B)** Quantification of bacterial CFUs of 24 flies was performed by homogenizing flies in PBS and plating serial dilutions on tryptic soy agar plates supplemented with sheep's blood and streptomycin. Each plate contains serial dilutions from 8 flies, with a total of 24 flies and 3 plates used per treatment. Image shows plates for 0-hour *S.p.* **C)** Image shows plates for 0-hour *S.p* + 250 µM LA. **D)** Image shows plates for 0-hour *S.p.* + 250 µM AA.

Abbreviation	Fatty acid	Abbreviation	Fatty acid
C18:1 cis	Oleic acid	C20:5	11-HEPE
C18:2	12,13-DiHOME	C20:4	tetranor-12-HETE
C18:2	12,13-EpOME-cis	C20:5	11,12-DiHETE
C18:2	13-HODE	C20:5	11,12-EEQ-cis
C18:2	13-oxo-ODE	C20:5	12-HEPE
C18:2	9,10-DiHOME	C20:5	14,15-DiHETE
C18:2	9,10-EpOME-cis	C20:5	14,15-EEQ-cis
C18:2	9-HODE	C20:5	15-HEPE
C18:2	9-oxo-ODE	C20:5	17,18-DiHETE
C18:3 alpha	9-oxo-OtrE	C20:5	17,18-EEQ-cis
C18:3 alpha	9-HOTrE	C20:5	18-HEPE
C18:3 n-3	alpha linolenic acid	C20:5	19-HEPE
C18:3 gamma	13-gamma-HOTrE	C20:5	20-HEPE
C20:3	12-HeTrE	C20:5	5,12-DiHETE
C20:3	14,15-EpEDE-cis	C20:5	5,6-DiHETE
C20:3	15-HeTrE	C20:5	5,6-EEQ
C20:3	5-HeTrE	C20:5	5-HEPE
C20:3	8-HeTrE	C20:5	8,9-DiHETE
C20:3	LTB3	C20:5	8,9-EEQ-cis
C20:4	11,12-DHET	C20:5	8-HEPE
C20:4	11,12-EET-cis	C20:5	9-HEPE
C20:4	11-HETE	C20:5	LTB5
C20:4	12-HETE	C22:6	10,11-DiHDPA
C20:4	12-HHTrE	C22:6	10,11-EDP-cis
C20:4	12-HpETE	C22:6	10-HDHA
C20:4	12-oxo-ETE	C22:6	11-HDHA
C20:4	14,15-DHET	C22:6	13,14-DiHDPA
C20:4	14,15-EET-cis	C22:6	13,14-EDP-cis
C20:4	15-HETE	C22:6	13-HDHA
C20:4	15-oxo-ETE	C22:6	14-HDHA
C20:4	16-HETE	C22:6	16,17-DiHDPA
C20:4	17-HETE	C22:6	16,17-EDP-cis
C20:4	18-HETE	C22:6	16-HDHA
C20:4	19-HETE	C22:6	17-HDHA
C20:4	20-COOH-ARA	C22:6	19,20-DiHDPA
C20:4	20-HETE	C22:6	19,20-EDP-cis
C20:4	5(S),15(S)-DiHETE	C22:6	20-HDHA
C20:4	5,6-EET	C22:6	21-HDHA
C20:4	5-HETE	C22:6	22-HDHA
C20:4	5-oxo-ETE	C22:6	4-HDHA
C20:4	6-trans-epi-LTB4	C22:6	4-oxo-DHA
C20:4	6-trans-LTB4	C22:6	7,8-DiHDPA
C20:4	8(S),15(S)-DiHETE	C22:6	7,8-EDP-cis
C20:4	8,9-DHET	C22:6	7-HDHA
C20:4	8,9-EET-cis	C22:6	8-HDHA
C20:4	8-HETE	C22:6	NPD-1
C20:4	9-HETE	C18:2	Linoleic acid
C20:4	LTB4	C20:4	Arachidonic acid

Supplemental Table 1: List of all fatty acids and oxylipins screened at 12 hours post-infection (Figure S1). A total of 96 metabolites were screened in the fatty acid and oxylipin panel by Lipotype GmbH. A total of

13 of the 96 metabolites were detected (indicated in red).

Fatty acid Abbreviation	Common name
12:0	Lauric Acid
14:0	Myristic Acid
15:0	Pentadecylic acid
16:0	Palmitic Acid
16:1	cis-9-palmitoleic acid
17:0	Margaric acid
17:1	Margaroleic acid
18:0	Stearic Acid
18:1	Oleic Acid
18:2	Linoleic acid
18:3 N3	alpha-Linolenic acid
18:3 N6	gamma-Linolenic acid
18:4	Stearidonic acid
20:0	Arachidic acid
20:1	cis-Gadoleic acid
20:2	11,14-eicosadienoic acid
20:3 N3	Dihomo-alpha-linolenic acid
20:3 N6	bishomo-gamma-linolenic acid
20:3 N9	5,8,11-eicosatrienoic acid (n9)
20:4	Arachidonic acid
20:5	Eicosapentaenoic acid (n3)
22:0	Behenic acid
22:1	cis-erucic acid
22:2	13Z,16Z-docosadienoic acid
22:3	DTrE
22:4	Adrenic Acid
22:5 N3	7,10,13,16,19-docosapentaenoic acid (n3)
22:5 N6	4,7,10,13,16-docosapentaenoic acid (n6)
22:6	Docosahexaenoic acid (n3)
23:0	Tricosylic acid
24:0	Lignoceric acid
24:1	Nervonic acid
26:0	Cerotic acid

Supplemental Table 2: List of all fatty acids screened in Figure 1. A total of 33 fatty acids were screened in the GC/MS fatty acid panel by UC San Diego Lipidomics Core. A total of 22 of the 33 metabolites were detected (indicated in red).

Name	Systemic Name	Name	Systemic Name	Name	Systemic Name	Name	Systemic Name
PGE2	9-oxo-11R,15S-dihydroxy-5Z,13E-prostadienoic acid; Prostin E2	8-iso-PGF2a	9S,11R,15S-trihydroxy-5Z,13E-prostadienoic acid-cyclo[8S,12R]	Protectin DX	10S,17S-dihydroxy-4Z,7Z,11E,13Z,15E,19Z-docosahexaenoic acid	(+/-)8,9-EpETrE	8,9-epoxy-5Z,11Z,14Z-eicosatrienoic acid
PGD2	9S,15S-dihydroxy-11-oxo-5Z,13E-prostadienoic acid	9-HETE	9-hydroxy-5Z,7E,11Z,14Z-eicosatetraenoic acid	8,15-DiHETE	(5Z,9E,11Z,13E)-8,15-dihydroxyicosa-5,9,11,13-tetraenoic acid	(+/-)11,12-EpETrE	11,12-epoxy-5Z,8Z,14Z-eicosatrienoic acid
11β-PGF2α	9S,11S,15S-trihydroxy-5Z,13E-prostadienoic acid	(+/-) 9-HEPE	(+/-)-9-hydroxy-5Z,7E,11Z,14Z,17Z-eicosapentaenoic acid	15S-HETE	15S-hydroxy-5Z,8Z,11Z,13E-eicosatetraenoic acid	(+/-)14,15-EpETrE	14,15-epoxy-5Z,8Z,11Z-eicosatrienoic acid
TXB1	9S,11,15S-trihydroxy-thrombox-13E-enoic acid	(+/-)-8-HDoHE	(+/-)-8-hydroxy-4Z,6E,10Z,13Z,16Z,19Z-docosahexaenoic acid	(+/-) 15-HEPE	(+/-)-15-hydroxy-5Z,8Z,11Z,13E,17Z-eicosapentaenoic acid	14(15)-EpETE	(+/-)-14(15)-epoxy-5Z,8Z,11Z,17Z-eicosatetraenoic acid
PGF1a	9S,11R,15S-trihydroxy-13E-prostaenoic acid	(+/-)-16-HDoHE	(+/-)-16-hydroxy-4Z,7Z,10Z,13Z,17E,19Z-docosahexaenoic acid	(+/-)-17-HDoHE	(+/-)-17-hydroxy-4Z,7Z,10Z,13Z,15E,19Z-docosahexaenoic acid	17(18)-EpETE	(+/-)-17(18)-epoxy-5Z,8Z,11Z,14Z-eicosatetraenoic acid
PGE1	9-oxo-11R,15S-dihydroxy-13E-prostaenoic acid	(+/-)-20-HDoHE	(+/-)-20-hydroxy-4Z,7Z,10Z,13Z,16Z,18E-docosahexaenoic acid	13(R)-HODE	13R-hydroxy-9Z,11E-octadecadienoic acid	16(17) EpDPE	(+/-)-16(17)-epoxy-4Z,7Z,10Z,13Z,19Z-docosapentaenoic acid
PGD1	9-oxo-15S,19-dihydroxy-5Z,8(12),13E-prostatrienoic acid	LTB4	5S,12R-dihydroxy-6Z,8E,10E,14Z-eicosatetraenoic acid	13(S)-HODE	13S-hydroxy-9Z,11E-octadecadienoic acid	19(20)-EpDPE	(+/-)-19(20)-epoxy-4Z,7Z,10Z,13Z,16Z-docosapentaenoic acid
TXB3	9S,11,15S-trihydroxy-thromboxa-5Z,13E,17Z-trien-1-oic acid	20-hydroxy LTB4	5S,12R,20-trihydroxy-6Z,8E,10E,14Z-eicosatetraenoic acid	13(S)-HOTrE	13S-hydroxy-9Z,11E,15Z-octadecatrienoic acid	19,20-DiHDPE	(+/-)-19,20-dihydroxy-4Z,7Z,10Z,13Z,16Z-docosapentaenoic acid
PGF3a	9S,11R,15S-trihydroxy-5Z,13E,17Z-prostatrienoic	20-carboxy-LTB4	5S,12R-dihydroxy-6Z,8E,10E,14Z-eicosatetraene-1,20-dioic acid	13(S)-HOTrE(γ)	13S-hydroxy-6Z,9Z,11E-octadecatrienoic acid	9(10)-EpOME	9,10-epoxy-12Z-octadecenoic acid
PGE3	9-oxo-11R,15S-dihydroxy-5Z,13E,17Z-prostatrienoic acid	5S,6R-DiHETE	5S,6R-dihydroxy-7E,9E,11Z,14Z-eicosatetraenoic acid	15(S)-HETrE	15S-hydroxy-8Z,11Z,13E-eicosatrienoic acid	12(13)-EpOME	(+/-)-12(13)-epoxy-9Z-octadecenoic acid
PGD3	9S,15S-dihydroxy-11-oxo-5Z,13E,17Z-prostatrienoic acid	5S,6S-DiHETE	5S,6S-dihydroxy-7E,9E,11Z,14Z-eicosatetraenoic acid	8S-HETE	8S-hydroxy-5Z,9E,11Z,14Z-eicosatetraenoic acid	(+/-)5,6-DiHETrE	5,6-dihydroxy-8Z,11Z,14Z-eicosatrienoic acid
1a,1b-dihomo-PGF2a	1a,1b-dihomo-9S,11R,15S-trihydroxy-5Z,13E-prostadienoic acid	5,12-DiHETE	5,12-dihydroxy-6,8,10,14-eicosatetraenoic acid	(+/-) 8-HEPE	(+/-)-8-hydroxy-5Z,9E,11Z,14Z,17Z-eicosapentaenoic acid	(+/-)8,9-DiHETrE	8,9-dihydroxy-5Z,11Z,14Z-eicosatrienoic acid
15-keto-PGF2α	9S,11R-dihydroxy-15-oxo-5Z,13E-prostadienoic acid	12-oxo-LTB4	5S-hydroxy-12-keto-6Z,8E,10E,14Z-eicosatetraenoic acid	(+/-)-10-HDoHE	(+/-)-10-hydroxy-4Z,7Z,11E,13Z,16Z,19Z-docosahexaenoic acid	(+/-)11,12-DiHETrE	11,12-dihydroxy-5Z,8Z,14Z-eicosatrienoic acid
15-keto-PGE2	9,15-dioxo-11R-hydroxy-5Z,13E-prostadienoic acid	LTC4	5S-hydroxy,6R-(S-glutathionyl),7E,9E,11Z,14Z-eicosatetraenoic acid	8(S)-HETrE	8S-hydroxy-9E,11Z,14Z-eicosatrienoic acid	(+/-)14,15-DiHETrE	14,15-dihydroxy-5Z,8Z,11Z-eicosatrienoic acid
13,14-dihydro-15-keto-PGF2α	9S,11S-dihydroxy-15-oxo-5Z-prostenoic acid	LTD4	5S-hydroxy-6R-(S-cysteinylglycinyl)-7E,9E,11E,14Z-eicosatetraenoic acid	12R-HETE	12R-hydroxy-5Z,8Z,10E,14Z-eicosatetraenoic acid	9,10-di-HOME	9(10)-dihydroxy-12Z-octadecenoic acid
13,14-dihydro-15-keto-PGE2	9,15-dioxo-11R-hydroxy-5Z-prostenoic acid	LTE4	5S-hydroxy,6R-(S-cysteinyl),7E,9E,11Z,14Z-eicosatetraenoic acid	12S-HETE	12S-hydroxy-5Z,8Z,10E,14Z-eicosatetraenoic acid	12,13-DiHOME	12,13-dihydroxy-9Z-octadecenoic acid
13,14-dihydro-15-keto-PGD2	11,15-dioxo-9S-hydroxy-5Z-prostenoic acid	11-trans-LTC4	5S-hydroxy-6R-(S-glutathionyl)-7E,9E,11E,14Z-eicosatetraenoic acid	(+/-) 12-HEPE	(+/-)-12-hydroxy-5Z,8Z,10E,14Z,17Z-eicosapentaenoic acid	Arachidonic acid	5Z,8Z,11Z,14Z-eicosatetraenoic acid
bicyclo-PGE2	9,15-dioxo-5Z-prostaenoic acid-cyclo[11S,16]	11-trans-LTD4	5S-hydroxy-6R-(S-cysteinylglycinyl)-7E,9E,11E14Z-eicosatetraenoic acid	(+/-)-14-HDoHE	(+/-)-14-hydroxy-4Z,7Z,10Z,12E,16Z,19Z-docosahexaenoic acid	Adrenic acid	7,10,13,16-docosatetraenoic acid
20-hydroxy-PGF2a	9S,11S,15S,20-tetrahydroxy-5Z,13E-prostadienoic acid	11-trans-LTE4	5S-hydroxy-6R-(S-cysteinyl)-7E,9E,11E14Z-eicosatetraenoic acid	(+/-)-11-HDoHE	(+/-)-11-hydroxy-4Z,7Z,9E,13Z,16Z,19Z-docosahexaenoic acid	EPA	5Z,8Z,11Z,14Z,17Z-eicosapentaenoic acid
20-hydroxy-PGE2	9-oxo-11R,15S,20-trihydroxy-5Z,13E-prostadienoic acid	5S-HETE	5S-hydroxy-6E,8Z,11Z,14Z-eicosatetraenoic acid	9(R)-HODE	9R-hydroxy-10E,12Z-octadecadienoic acid	DHA	4Z,7Z,10Z,13Z,16Z,19Z-docosahexaenoic acid
2,3-dinor 11β-PGF2a	9S,11S,15S-trihydroxy-2,3-dinor-5Z,13E-prostadienoic acid	(+/-) 5-HEPE	(+/-)-5-hydroxy-6E,8Z,11Z,14Z,17Z-eicosapentaenoic acid	9(S)-HODE	9S-hydroxy-10E,12Z-octadecadienoic acid	20-carboxy-AA	5Z,8Z,11Z,14Z-Eicosatetraenedioic acid
tetranor-PGFM	9S,11R-dihydroxy-15-oxo-2,3,4,5-tetranor-prostan-1,20-dioic acid	(+/-)-7-HDoHE	(+/-)-7-hydroxy-4Z,8E,10Z,13Z,16Z,19Z-docosahexaenoic acid	5-Oxo-ETE	5-oxo-6E,8Z,11Z,14Z-eicosatetraenoic acid	17-oxo-DPA	7Z,10Z,13Z,15E,19Z-17-oxo-docosapentaenoic acid
Tetranor-PGEM	11R-hydroxy-9,15-dioxo-2,3,4,5-tetranor-prostan-1,20-dioic acid	(+/-)-4-HDoHE	(+/-)-4-hydroxy-5E,7Z,10Z,13Z,16Z,19Z-docosahexaenoic acid	12-oxo-ETE	12-oxo-5Z,8Z,10E,14Z-eicosatetraenoic acid	2,3-Dinor-TXB2	9S,11,15S-trihydroxy-2,3-dinor-thromboxa-5Z,13E-dien-1-oic acid
Tetranor-12(R)-HETE	8R-hydroxy-4Z,6E,10Z-hexadecatrienoic acid	9(S)-HOTrE	9S-hydroxy-10E,12Z,15Z-octadecatrienoic acid	15-Oxo-ETE	15-oxo-5Z,8Z,11Z,13E-eicosatetraenoic acid	11-dehydro-TXB2	9S,15S-dihydroxy-11-oxo-thromboxa-5Z,13E-dien-1-oic acid
PGK2	9,11-dioxo-15S-hydroxy-5Z,13E-prostadienoic acid	5(S)-HETrE	5S-hydroxy-6E,8Z,11Z-eicosatrienoic acid	9-OxoODE	9-oxo-10E,12Z-octadecadienoic acid	2,3-Dinor-8-iso-PGF2alpha	9S,11R,15S-trihydroxy-2,3-dinor-5Z,13E-prostadienoic acid-cyclo[8S,12R]
12S-HHTrE	12S-hydroxy-5Z,8E,10E-heptadecatrienoic acid	5S,15S-DiHETE	5S,15S-dihydroxy-6E,8Z,11Z,13E-eicosatetraenoic acid	13-OxoODE	13-keto-9Z,11E-octadecadienoic acid	2,3-dinor, 6-keto-PGF1a	6-oxo-9S,11R,15S-trihydroxy-2,3-dinor-13E-prostaenoic acid
11S-HETE	11S-hydroxy-5Z,8Z,11E,14Z-eicosatetraenoic acid	5S,6R-LipoxinA4	5S,6R,15S-trihydroxy-7E,9E,11Z,13E-eicosatetraenoic acid	15-Oxo-EDE	15-oxo-11Z,13E-eicosadienoic acid	8-iso-PGF3alpha	9S,11R,15S-trihydroxy-5Z,13E,17Z-prostatrienoic acid-cyclo[8S,12R]
(+/-) 11-HEPE	(+/-)-11-hydroxy-5Z,8Z,12E,14Z,17Z-eicosapentaenoic acid	15-epi-lipoxin A4	(5S,6R,7E,9E,11Z,13E,15R)-5,6,15-trihydroxyicosa-7,9,11,13-tetraenoic acid	20-HETE	20-hydroxy-5Z,8Z,11Z,14Z-eicosatetraenoic acid	8-iso-15-keto Prostaglandin F2β	9β,11α-dihydroxy-15-oxo-(8β)-prosta-5Z,13E-dien-1-oic acid
(+/-)-13-HDoHE	(+/-)-13-hydroxy-4Z,7Z,10Z,14E,16Z,19Z-docosahexaenoic acid	epi-Lipoxin A4	5S,6S,15S-trihydroxy-7E,9E,11Z,13E-eicosatetraenoic acid	19-HETE	(5Z,8Z,11Z,14Z)-19-hydroxy-5,8,11,14-icosatetraenoic acid	9-Nitrooleate	9-nitro-9E-octadecenoic acid
PGA2	9-oxo-15S-hydroxy-5Z,10Z,13E-prostatrienoic acid	LXA5	5S,6R,15S-trihydroxy-7E,9E,11Z,13E,17Z-eicosapentaenoic acid	18-HETE	18-hydroxy-5Z,8Z,11Z,14Z-eicosatetraenoic acid	10-Nitrooleate	10-nitro-9E-octadecenoic acid
PGB2	15S-hydroxy-9-oxo-5Z,8(12),13E-prostatrienoic acid	Lipoxin B4	5S,14R,15S-trihydroxy-6E,8Z,10E,12E-eicosatetraenoic acid	17-HETE	17-hydroxy-5Z,8Z,11Z,14Z-eicosatetraenoic acid	tetranor-PGDM	9S-hydroxy-11,15-dioxo-2,3,4,5-tetranor-prostan-1,20-dioic acid
PGJ2	11-oxo-15S-hydroxy-5Z,9,13E-prostatrienoic acid	Resolvin-E1	5S,12R,18R-trihydroxy-6Z,8E,10E,14Z,16E-eicosapentaenoic acid	16-HETE	(5Z,8Z,11Z,14Z)-16-hydroxyicosa-5,8,11,14-tetraenoic acid	Maresin 1	7R,14S-dihydroxy-4Z,8E,10E,12Z,16Z,19Z-docosahexaenoic acid
15-deoxy-delta-12,14-PGD2	9S-hydroxy-11-oxo-5Z,12E,14E-prostatrienoic acid	Resolvin-D1	7S,8R,17S-trihydroxy-4Z,9E,11E,13Z,15E,19Z-docosahexaenoic acid	(+/-) 18-HEPE	(+/-)-18-hydroxy-5Z,8Z,11Z,14Z,16E-eicosapentaenoic acid	Resolvin D2	7S,16R,17S-trihydroxy-4Z,8E,10Z,12E,14E,19Z-docosahexaenoic acid
15-deoxy-δ-12,14-PGJ2	11-oxo-5Z,9,12E,14E-prostatetraenoic acid	7(S),17(S)-hydroxy DPA	,17-dihydroxy-8E,10Z,13Z,15E,19Z-docosapentaenoic acid	(+/-)5,6-EpETrE	5,6-epoxy-8Z,11Z,14Z-eicosatrienoic acid	Resolvin D3	4S,10,17S-trihydroxy-5E,7E,9E,13Z,15E,19Z-docosahexaenoic acid
						Resolvin D5	7S,17S-dihydroxy-4Z,8E,10Z,13Z,15E,19Z-docosahexaenoic acid

Supplemental Table 3: List of all eicosanoids screened in Figure 2. A total of 141 eicosanoids were screened in the LC/MS eicosanoid panel by UC San Diego Lipidomics Core. A total of 42 of the 141 metabolites were detected (indicated in red).

Primer	Sequence 5'-3'	Gene
Tub_fwd<br>Tub_rvs	TCCACTCGTTCGGTGGAGGT<br>GGGCTGGGTAGATGGCGAAC	FBgn0003884
Dros_fwd<br>Dros_rvs	TGTTCGCCCTCTTCGCTGTC<br>CTGGAGCGTCCCTCCTCCTT	FBgn0283461
Def_fwd<br>Def_rvs	CAGGCTCAGCCAGTTTCCGA<br>TCGCATGTGGCTCGCTTCTG	FBgn0010385
Metch_fwd<br>Metch_rvs	ATGCAACTTAATCTTGGAGCGA<br>TGTGTTAACGACATCAGCAGTGTG	FBgn0014865
Dipt_fwd<br>Dipt_rvs	CGTCGCCTTACTTTGCTGC<br>CCCTGAAGATTGAGTGGGTACTG	FBgn0004240

Supplemental Table 4: Primers used for AMP expression by qPCR.
